# Two cases of concurrent carcinoma showing thymus-like differentiation (CASTLE) coexisting with papillary thyroid carcinoma

**DOI:** 10.1093/jscr/rjad527

**Published:** 2023-09-24

**Authors:** Qi Zhao, Xuehai Bian

**Affiliations:** Department of Thyroid Surgery, China–Japan Union Hospital of Jilin University, Jilin Provincial Key Laboratory of Surgical Translational Medicine, Changchun 130033, China; Department of Thyroid Surgery, China–Japan Union Hospital of Jilin University, Jilin Provincial Key Laboratory of Surgical Translational Medicine, Changchun 130033, China

**Keywords:** carcinoma showing thymus-like differentiation (CASTLE), papillary thyroid carcinoma, diagnosis, neck dissection, radiotherapy

## Abstract

Carcinoma showing thymus-like differentiation (CASTLE), which emerges within the thyroid gland or the adjacent soft tissues of the neck, is a rare malignant neoplasm found globally. The occurrence of CASTLE in conjunction with papillary thyroid carcinoma is an even more infrequent phenomenon. The ensuing sections elaborate upon the clinical attributes characteristic of CASTLE.

## Introduction

Carcinoma showing thymus-like differentiation (CASTLE) of the thyroid constitutes a malignancy characterized by thymic epithelial differentiation. Up until the present time, a mere five instances of such occurrences have been documented in the body of English literature [1–5]. Initially reported by Miyauchi *et al*. [6] as an intrathyroidal epithelial thymoma, its histological similarity to thymic carcinoma, lymphoepithelioma-like carcinoma, or squamous cell carcinoma was subsequently discerned by Chan and Rosai [7]. They proposed that it arises from ectopic thymic tissues or remnants of branchial pouches, retaining the potential for thymic differentiation. Consequently, they renamed it CASTLE. Even though CASTLE typically exhibits relatively indolent biological behavior and a generally favorable prognosis, its clinical presentation and histopathology resemble thyroid squamous cell carcinoma, undifferentiated carcinoma, etc. This resemblance often results in misdiagnosis and the potential for unnecessary aggressive treatments. Hence, achieving a precise diagnosis and making informed decisions about treatment, which encompass surgical approaches and postoperative strategies, are crucial in ensuring the optimal prognosis for these patients. In this article, we present the sixth and seventh occurrences of this nature and compare their clinical case characteristics with the previously reported five cases, as summarized in Table 1.

**Table 1:** The clinical characteristics, treatments, and outcomes of the patients.

Cases	Watanabe *et al.*	Kimura *et al.*	Hsu *et al.*	Wu *et al.*	Kwak *et al.*	This case 1	This case 2
Age/sex	32/female	66/male	58/female	44/male	50/male	41/female	38/male
PTC							
Tumor size	diam.0.8 cm	diam.0.5 cm	1.8 × 2.4 cm	0.56 × 0.42 cm	unknown	0.8 × 0.6 cm	diam.0.1 cm
location	up.of rl.	lp.of rl.	lp.of rl.	rl.	rl.	ll.；rl.	up.of rl.
CASTLE							
Tumor size	4.5 × 3.5 cm	1.5 × 2.3 × 2.5 cm	unknown	4.8 × 5.5 cm	5 × 4 × 4 cm	3.19 × 1.9 cm	4.12 × 2.43 cm
location	lp.of ll.	lp.of rl.	lp.of rl.	ll.	rl.	lp.of rl.	lp.of rl.
LN biopsy	No tumor	LN metastasis	No tumor	unknown	unknown	LN metastasis	No tumor
Treatment	total thyroidectomy with neck dissection	total thyroidectomy with neck dissection of the central area and right lateral cervical lymph nodes	total thyroidectomy and CND	total thyroidectomy	total thyroidectomy with neck dissection of the central area and right lateral cervical lymph nodes	total thyroidectomy and aCND	Lobectomy with CND
Outcome	local recurrence after 5 months	NED after 5 years	NED	NED after 5 years	NED	NED after 12 months	NED after 5 years

## Case report

### Case 1

**Figure 1:**
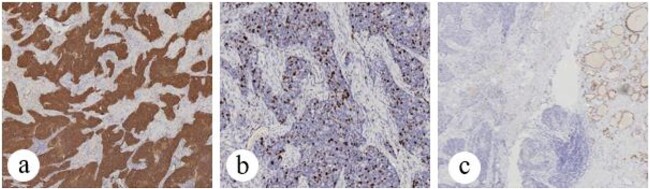
Patient no. 1 (a) tumor cells are positive for CK5/6, (b) tumor cells showed 20% positivity for Ki67, SP × 10, and (c) tumor cells showed negativity for TTF-1, SP × 4.

A 41-year-old female presented with a 1-year history of a mass in the lower right part of her neck. Upon physical examination, a solitary, non-tender, and relatively fixed mass was detected in the right superior sternal fossa. Sonography indicated the presence of a hypoechoic nodule measuring 0.90 × 0.94 cm with an indistinct boundary situated in the central and lower segment of the left thyroid lobe. Additionally, a similar hypoechoic nodule measuring 0.73 × 0.70 cm was observed in the central region of the right lobe. Furthermore, a hypoechoic mass measuring 3.19 × 1.9 cm, characterized by the absence of calcification but displaying moderate vascularity, was identified in the lower end of the right thyroid lobe. Subsequent cervical CT scans unveiled a soft-tissue mass of 3.2 × 1.7 cm dimensions, exhibiting an indistinct perimeter. The utilization of thyroid scintigraphy involving 131I demonstrated a hypo-functioning region in the lower region of the right lobe. Subsequently, fine-needle aspiration cytology (FNAC) was executed, and the assessment of nodules in the left lobe and the central area of the right lobe revealed an atypia. The resulting diagnosis was reported as “papillary thyroid carcinoma” (PTC). However, the biopsy diagnosis for the mass located in the inferior pole of the right thyroid lobe was determined as “Suspicious of thyroid carcinoma of unknown nature.” Routine blood investigations and thyroid function tests yielded results within the normal ranges. Additionally, a core needle biopsy (CNB) procedure was conducted, disclosing clusters of malignant tumor cells arranged in a nest-like pattern. When coupled with immunohistochemical analysis (which showed positivity for CK5/6, P63, CD5, CD117, slight positivity for Syn, 20% positivity for Ki67, and negativity for Vim, TTF-1, S-100, and CgA), a diagnosis of “carcinoma of thymic origin could not be excluded” was made (Fig. 1). During the surgical procedure, a tumor measuring 3.0 × 2.0 cm was observed situated in the lower segment of the right thyroid lobe. The tumor extended across the trachea and infiltrated the adjacent soft tissue. A total thyroidectomy with central neck dissection was performed. Subsequent to this, the conclusive pathology report confirmed the presence of PTC in both the left lobe (0.8 × 0.6 cm in size) and right lobe (with a diameter of 0.1 cm). Additionally, the mass situated in the lower portion of the right lobe was identified as “CASTLE,” a diagnosis supported by consistent immunohistochemical outcomes as mentioned previously. Metastasis was detected in a single lymph node out of a total of 20 nodes examined. No radiotherapy or chemotherapy was administered, but TSH suppression therapy was initiated after the operation. After 3 years of post-surgery follow-up, there were no signs of recurrent or metastatic disease.

### Case 2

**Figure 2:**
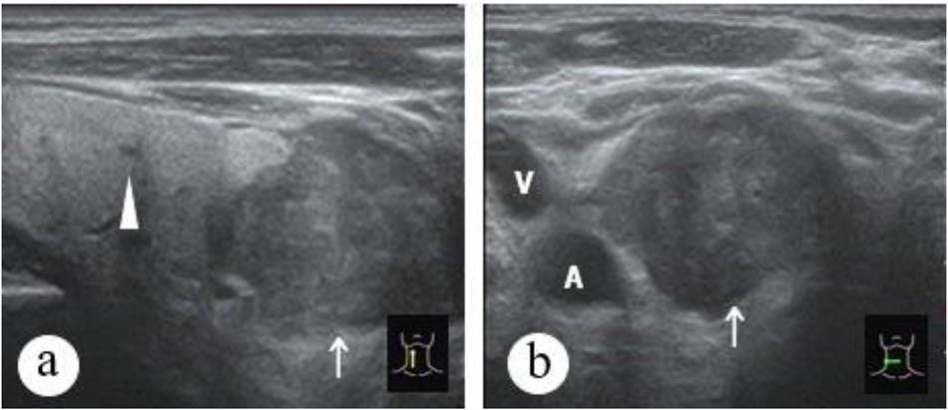
Patient no. 2 (a) thyroid ultrasonography image shows an uneven hypoechoic mass of the tumor, with multiple bands of high echogenicity intersecting inside. CASTLE [→], PTC(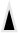) and (b) the positional relationship between the tumor and the arteriovenous system.

**Figure 3:**
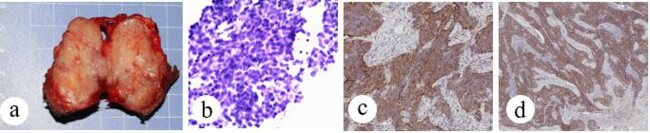
Patient no. 2 (a) operative specimen, (b) FNAC shows nest-like small round tumor cells, (c) tumor cells are positive for CD5, SP × 10, and (d) tumor cells are positive for CD117, SP × 4.

During a routine health examination 3 months ago, a 39-year-old man was incidentally discovered to have an asymptomatic mass in the anterior lower right side of his neck. The patient has no comorbidities or family history of neoplastic diseases. Thyroid ultrasonography revealed the presence of an inhomogeneous and low echogenic mass measuring 4.2 × 2.6 × 2.2 cm in the right lobe of the thyroid (Fig. 2a). Furthermore, a tiny nodule with low echogenicity, measuring 0.28 × 0.20 cm, was identified in the superior right portion of the thyroid gland. No abnormal cervical lymph nodes were detected in the sonographic examinations. The laboratory study results were within the normal range. FNAC of the inferior mass revealed the presence of nest-like small round tumor cells within fibrous proliferated tissues under microscopy, indicating the possibility of a malignant tumor (Fig. 3b). Lobectomy with isthmectomy, along with ipsilateral central cervical lymph node dissection, was performed (Fig. 3a). Under microscopic analysis, cords and nests of epithelial cells were observed, and there was some noticeable squamous differentiation in specific areas. Immunohistochemical analysis indicated positive staining for CD5 (Fig. 3c), CD117 (Fig. 3d), Ki-67 (20%+), P63, P40, and p53 (30%+), whereas negative staining was observed for Tg, TTF-1, and BRAF. Considering these observations, the primary tumor was diagnosed as “CASTLE,” whereas the nodule in the upper pole was diagnosed as PTC with a diameter of 0.1 cm. Examination of the central neck region revealed no signs of metastasis (0/6 lymph nodes). The patient received TSH suppression therapy but did not undergo radiotherapy or chemotherapy. After 12 months of post-surgery follow-up, there were no signs of recurrent or metastatic disease.

## Discussion

Thyroid CASTLE is a type of primary thyroid cancer characterized by histological and immunohistochemical attributes that bear a resemblance to thymic epithelial tumors. Presently, the prevailing viewpoint among scholars is that CASTLE likely emerges from ectopic thymic tissues or residual tissues from branchial pouches. This particular type of thyroid carcinoma is more commonly found in middle-aged women aged 40 and above. Most individuals affected by this condition typically exhibit painless and gradual enlargement of neck masses as the primary symptom. In a smaller subset of cases, patients might encounter compression-related symptoms at the time of diagnosis, such as hoarseness, challenges in swallowing, and breathing issues. CASTLE is commonly located in the lower pole of the thyroid gland, with only a few reported cases of extrathyroidal invasion. In this case, ultrasound imaging revealed a significant, heterogeneous hypoechoic mass with multiple internal echogenic septations, consistent with the findings reported in the literature [8].

The preoperative and intraoperative diagnosis of CASTLE poses challenges. Preoperative imaging frequently leads to confusion with thyroid adenomas, and intraoperative pathological assessments might incorrectly identify it as a poorly differentiated carcinoma. Consequently, establishing a conclusive diagnosis necessitates histopathological examination and immunohistochemical staining of tissues embedded in paraffin. Microscopic examination of this case revealed tumor cells arranged in island-like and cord-like patterns, with certain regions showing indications of squamous differentiation. Immunohistochemical staining revealed positive expression of CD5 and CD117 in tumor cells and negative expression of Tg and TTF-1. These findings are consistent with the typical immunohistochemical patterns reported in the literature. CD5 is the most commonly used antibody for diagnosing CASTLE. In the study by Ito *et al*. [9], the sensitivities and specificities of CD5 diagnosis were noted to be 82% and 100%, respectively. While CD5-negative expression is rare in CASTLE, CD117 staining should be considered in such cases. It is essential to distinguish CASTLE from more aggressive and negatively prognostic malignancies like poorly differentiated and undifferentiated thyroid carcinomas, primary thyroid squamous cell carcinomas, and metastatic carcinomas. Positive co-expression of both CD5 and CD117 in immunohistochemistry assists in distinguishing and classifying the condition [10].

At present, surgical removal is commonly regarded as the preferred approach for treating CASTLE. Choi *et al*. conducted a comprehensive analysis of existing literature, outlining the various treatment modalities, and corresponding outcomes of CASTLE. Their conclusion was that a more effective strategy than mere lesion resection involves a combined approach of neck lymph node dissection or postoperative radiotherapy. Research findings indicate that in cases where there is positive cervical lymph node metastasis, the use of adjuvant radiotherapy has been shown to decrease the recurrence rate significantly, from 100% to 57%. However, it is important to note that chemotherapy does not provide any beneficial impact on the prognosis in such situations [11, 12] In this case, the patient underwent TSH suppression therapy postoperatively because of concurrent PTC. Subsequent follow-up examinations revealed no signs of recurrence or metastasis. This article compiles information on reported cases of CASTLE in conjunction with PTC.

## Conclusion

In summary, when clinicians encounter slowly enlarging painless masses, especially those located at the lower pole of the thyroid gland, exhibiting ultrasonographic features such as lobulated, heterogeneous hypoechoic characteristics (occasionally with calcification), along with cytomorphological features indicative of malignancy that do not align with typical thyroid carcinoma characteristics, it is important to include CASTLE as a potential diagnosis. If the patient’s health permits, it is advisable to conduct a CNB along with immunohistochemical analysis. The presence of positive CD5 and CD117 staining in tumor cells, coupled with negative thyroid marker staining, can offer a preoperative diagnosis of CASTLE (as well as other thymic differentiated tumors). During surgery, it is crucial for surgeons to diligently pursue curative tumor resection by excising not only the tumor but also any adjoining tissues that the tumor has infiltrated. Comprehensive lymph node dissection is important, and the determination of whether to administer postoperative radiotherapy should be guided by factors such as adjacent tissue invasion and lymph node metastasis.

## Data Availability

The data underlying this article are available in the article and in its online supplementary material.

## References

[1] Kwak KH, Lee JG, Lee JK, Park JH. A case of carcinoma showing thymus-like differentiation (CASTLE) combined with papillary carcinoma in the thyroid. J Clin Otolaryngol Head Neck Surg 2015;26:307–11.

[2] Kimura T, Enomoto K, Kono M, et al. A case of concurrent occurrence of carcinoma showing thymus-like differentiation and follicular variant of papillary thyroid cancer in the same thyroid. J Surg Case Rep 2022;2022:rjab570. 35047168 10.1093/jscr/rjab570 PMC8760851

[3] Hsu YC, Hsueh C, Lin WN, et al. Carcinoma showing thymus-like differentiation (CASTLE) with synchronous papillary thyroid carcinoma: a case report and review. Ear Nose Throat J 2021;014556132110601. 10.1177/01455613211060167. 34866458

[4] Wu M, Luo M, Zhao Y et al.. Intrathyroid thymic carcinoma with papillary carcinoma of thyroid: a case report. 2022. https://www.researchsquare.com/article/rs-1616890/v1 10.21203/rs.3.rs-1616890/v1 (12 August 2023, date last accessed).

[5] Watanabe I, Tezuka F, Yamaguchi M, et al. Thymic carcinoma of the thyroid. Pathol Int 1996;46:450–6. 8869997 10.1111/j.1440-1827.1996.tb03636.x

[6] Miyauchi A, Kuma K, Matsuzuka F, et al. Intrathyroidal epithelial thymoma: an entity distinct from squamous cell carcinoma of the thyroid. World J Surg 1985;9:128–34. 3984364 10.1007/BF01656263

[7] Chan JKC, Rosal J. Tumors of the neck showing thymic or related branchial pouch differentiation: a unifying concept. Hum Pathol 1991;22:349–67. 2050369 10.1016/0046-8177(91)90083-2

[8] Yamamoto Y, Yamada K, Motoi N, et al. Sonographic findings in three cases of carcinoma showing thymus-like differentiation: sonographic images of castle. J Clin Ultrasound 2013;41:574–8. 23055246 10.1002/jcu.21997

[9] Ito Y, Miyauchi A, Nakamura Y, et al. Clinicopathologic significance of intrathyroidal epithelial thymoma/carcinoma showing thymus-like differentiation: a collaborative study with member Institutes of the Japanese Society of Thyroid Surgery. Am J Clin Pathol 2007;127:230–6. 17210519 10.1309/VM7E52B6U9Q729DQ

[10] Youens KE, Bean SM, Dodd LG, Jones CK. Thyroid carcinoma showing thymus-like differentiation (CASTLE): case report with cytomorphology and review of the literature. Diagn Cytopathol 2010;39:204–9. 10.1002/dc.21399 20607745

[11] Chan LP, Chiang FY, Lee KW, Kuo WR. Carcinoma showing thymus-like differentiation (CASTLE) of thyroid: a case report and literature review. Kaohsiung J Med Sci 2008;24:591–7. 19239992 10.1016/S1607-551X(09)70020-8 PMC11918237

[12] Gao R, Jia X, Ji T, et al. Management and prognostic factors for thyroid carcinoma showing thymus-like elements (CASTLE): a case series study. Front Oncol 2018;8:477. 30416983 10.3389/fonc.2018.00477 PMC6212596

